# Clarifications on: Pectoralis Blocks Nomenclature and Clinical Applications of Regional Anesthesia Techniques for Breast and Thorax

**DOI:** 10.5811/cpcem.1451

**Published:** 2023-11-30

**Authors:** Raghuraman M. Sethuraman

**Affiliations:** Sree Balaji Medical College & Hospital, BIHER, Department of Anesthesiology, Chennai, India

## To The Editor

I read with great interest the recently published case series applying pectoralis blocks (Pecs blocks) for infective breast conditions.1 I greatly commend Brewer et al for employing pecs blocks in the emergency department (ED) and hope many emergency physicians will adopt the interfascial plane blocks introduced in the last decade. I wish to provide a few clarifications regarding them.

Regarding nomenclature, the authors used the term “Pecs I and Pecs II”^1^ and attributed it to a “lack of consensus” and cited our article to support that.^2^ However, we categorically stated that it is incorrect to use that term (despite weak consensus); hence, I am surprised that Brewer et al used it throughout their article. I reiterate that stating “Pecs II block” (modified pecs block) itself is enough, as it is a combination of the Pecs I block (ie, interpectoral plane (IPP) block) and the pectoserattus plane (PSP) block. To make it simple, we must use the term either pecs II or IPP+PSP blocks. Otherwise, it defeats the very purpose of the suggestion of nomenclatures by the experts.^3^

Regarding the choice of the block, we must pay careful attention to the sensory coverage of each block. For instance, in my view the IPP block is not required for drainage of breast abscess as it provides only relief from myofascial pain due to the disruption of pectoral muscles and does not block the thoracic nerves that are involved in the sensory innervation of the breast.^4^ Also, there is a potential possibility of the presence of infection at the needle entry, precluding an IPP block in some cases. As infective conditions of the breast involve mainly the skin and subcutaneous tissues, either PSP block alone or its equivalent in sensory coverage, a serratus anterior plane (SAP) block, would be adequate. Of note, the SAP block is technically easier to perform compared to the PSP block. Also, the site of needle entry would be farther away from the infected tissues. Alternatively, I suggest a modification of the pecs block (“Pecs Zero”) introduced by Tulgar et al wherein the needle entry would be above the clavicle.^5^

Recently, the erector spinae plane (ESP) block has gained popularity as a promising technique for pain relief in the ED setting for various conditions.^6^ This block requires less expertise and time when compared to other fascial plane blocks. While Pecs blocks and the SAP block can be performed in the supine position, the ESP block requires a position other than supine such as sitting, lateral recumbent, or prone.

## References

[1] Brewer JH Sanders N Ayala A et al. The pectoralis block: A case series of a novel modality for acute pain control in the emergency department. Clin Pract Cases Emerg Med. 2023;7(2):60–3. 37285505 10.5811/cpcem.1408 PMC10247170

[2] Sethuraman RM Narayanan V. Pecs II block: Clarifications sought on nomenclature. Reg Anesth Pain Med. 2022;47(7):450. 10.1136/rapm-2022-103623 35443994

[3] El-Boghdadly K Wolmarans M Stengel AD et al. Standardizing nomenclature in regional anesthesia: an ASRA-ESRA Delphi consensus study of abdominal wall, paraspinal, and chest wall blocks. Reg Anesth Pain Med. 2021;46(7):571–80. 34145070 10.1136/rapm-2020-102451

[4] Woodworth GE Ivie RMJ Nelson SM et al. Perioperative breast analgesia: A qualitative review of anatomy and regional techniques. Reg Anesth Pain Med. 2017;42(5):609–31. 28820803 10.1097/AAP.0000000000000641

[5] Tulgar S Selvi O Thomas DT et al. A novel approach to blockage of pectoral nerves: Ultrasound guided modified clavipectoral fascial plane block (PECs-Zero). J Clin Anesth. 2020;59:49–50. 31220688 10.1016/j.jclinane.2019.06.024

[6] Abdelhamid K ElHawary H Turner JP. The use of the erector spinae plane block to decrease pain and opioid consumption in the emergency department: A literature review. J Emerg Med. 2020 Apr;58(4):603–9. 32245689 10.1016/j.jemermed.2020.02.022

